# A Magnetic Sensor Based on the Nonlinear Effect of Co-Rich Amorphous Wire

**DOI:** 10.3390/s22239117

**Published:** 2022-11-24

**Authors:** Dongfeng He

**Affiliations:** National Institute for Materials Science, 1-2-1 Sengen, Tsukuba 305-0047, Ibaraki, Japan; he.dongfeng@nims.go.jp; Tel.: +81-29-859-2533

**Keywords:** amorphous wire, magnetic sensor, FeCoSiB, inductive coil

## Abstract

A DC voltage induced by a DC magnetic field was observed for a coil with a Co-rich amorphous wire (FeCoSiB) as the core when an AC current flowed through the coil. The coil was 40 turns wound around a FeCoSiB amorphous wire with a diameter of 0.1 mm and a length of 8 mm. The magnitude of the DC voltage was determined by the frequency of the AC current, the amplitude of the AC current, and the applied DC magnetic field. When the sine wave current was 78 mA and the frequency was 6.8 MHz, a peak value of about 90 mV/Gauss DC voltage was observed. This phenomenon might have a relationship with the nonlinearity of the coil with the FeCoSiB amorphous wire as the core. A magnetic sensor with only an amplifier and a low-pass filter was developed using this effect.

## 1. Introduction

Due to their unique magnetic characteristics, Co-rich ferromagnetic amorphous wires, such as (Fe_0.06_Co_0.94_)_72.5_Si_2.5_B_15_ (FeCoSiB), have been fabricated and studied [1,2]. For amorphous wires with negative and nearly zero magnetostriction, the surface anisotropy is circular, and the inner anisotropy is perpendicular to the wire axis, which leads to the formation of a specific domain structure consisting of outer shell circular domains and inner core axial domains [3,4]. Several theoretical models, including the quasistatic model [5], eddy current model [6], domain model [7], electromagnetic model [8], and exchange-conductivity model [9,10], have been developed to explain the giant magnetoimpedance (GMI) effect of the amorphous wire.

Amorphous ferromagnetic wires exhibit very peculiar properties suitable for magnetic sensor applications [11,12,13]. Based on the special properties of the Co-rich ferromagnetic amorphous wire, pT-level high-sensitivity magnetic sensors have been developed using the coil with the FeCoSiB amorphous wire core.

**Figure 1:**
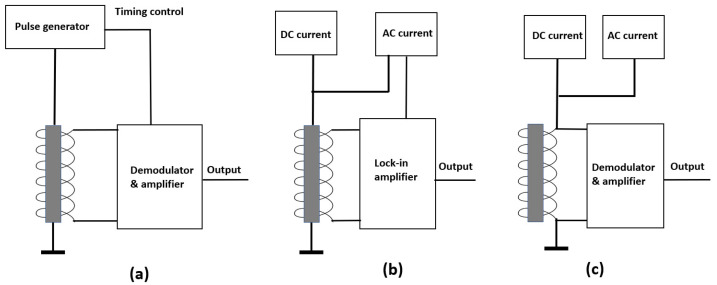
High-sensitivity magnetic sensors with Co-rich ferromagnetic amorphous wires (**a**). Block diagram of the GMI sensor (**b**). Block diagram of the orthogonal fluxgate sensor (**c**). Block diagram of the parallel fluxgate sensor.

Figure 1 shows three types of the high-sensitivity magnetic sensors with ferromagnetic amorphous wires. They were developed using the different effects of the amorphous wires, and the main differences of the magnetic sensors were their bias methods.

Figure 1a shows the schematic diagram of the GMI magnetic sensor [14,15]. The pulse generator produced a high-frequency square wave current sent to the amorphous wire. The coil was used to pick up the pulse signal modulated by the applied magnetic field. The frequencies of the pulse were normally from several tens of MHz to GHz [16,17]. Ultrahigh-sensitivity GMI sensors of the pT level were developed and used to measure the biomagnetic fields [18,19].

Figure 1b shows the schematic diagram of the orthogonal fluxgate sensor [20,21,22]. A DC current and an AC current were connected to the amorphous wire. The currents produced a circumferential excitation magnetic field around the amorphous wire, and the detection field was along the direction of the amorphous wire. The two directions were perpendicular, so this sensor was called an orthogonal fluxgate. For the orthogonal fluxgate, the frequency of the AC current was normally from tens of kHz to several hundreds of kHz and a pT-level high sensitivity was also achieved.

For both the GMI magnetic sensor and the orthogonal fluxgate sensor, there was an electrical connection between the driving circuit and the amorphous wire, which was not convenient for some applications, such as the magnetic microscope, so we developed a kind of parallel fluxgate sensor using the Co-rich amorphous wire shown in Figure 1c [23,24]. The DC current and AC current were connected to the coil instead of the amorphous wire. The direction of the excitation field was parallel with the detected magnetic field. For the parallel fluxgate sensor, the noise of the DC current and the AC current had a large influence on the total noise of the magnetic sensor, and the low-frequency performance was not as good as that of the GMI sensor or the orthogonal fluxgate [25]. For all three types of magnetic sensors with amorphous wires, a demodulator or lock-in amplifier must be used to measure the magnetic field.

In this paper, we presented the effect of the coil with the Co-rich FeCoSiB amorphous wire as the core, which was not previously reported. When an AC current flowed into the coil, a giant DC voltage was observed across the coil for the applied DC magnetic field. This “abnormal” DC voltage was not observed for a normal inductive coil without or with a normal ferrite core. This effect could be used to simplify the driving circuit of the magnetic sensor. Based on this effect, a magnetic sensor was developed. For a normal magnetic sensor with an amorphous wire, demodulation must be used in the driving circuit, but for this magnetic sensor, only an amplifier was used.

In Section 2, the properties of the FeCoSiB amorphous wire and the experimental methods were described. In Section 3, the experimental results of the DC voltage changing with the applied DC magnetic field as well as the frequency and the amplitude of the AC bias current were presented. Section 4 was a discussion.

## 2. Materials and Methods

The ϕ 0.1 mm FeCoSiB amorphous wire was fabricated using a water-quenched spinning method [26,27]. For this FeCoSiB amorphous wire with the ratio of Fe (0.06) and Co (0.94), the magnetostriction (the small change in dimension when a magnetic field is applied) value was close to zero [28], and the B-H curve had small hysteresis [29]. The saturation magnetization of the wire was about 0.81 T. The relative permeability was about 2000 near zero magnetic field.

**Figure 2:**
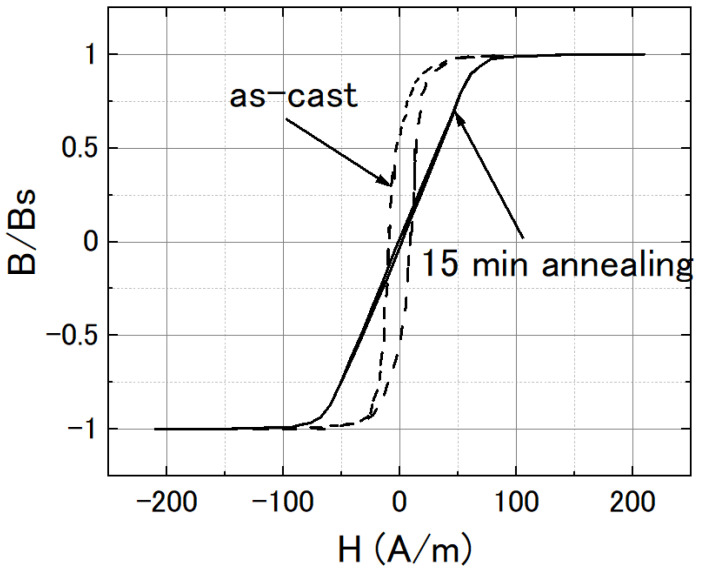
B-H loop of the FeCoSiB amorphous wire in its as-cast form (dashed line) and after 15 min of annealing (solid line).

To reduce the circumferential anisotropy and to reduce the 1/f noise of the magnetic sensor based on the amorphous wire, a Joule annealing method was used [30] by injecting about a 400 mA DC current into the amorphous wire. The temperature was increased to about 200 °C by Joule heating. An inductive method was used to measure the B-H curve of the amorphous wire [31]. The 5 mm long wire was placed in a 1 cm long solenoid, which created an axial magnetic field along the wire by applying a 30 Hz AC current to it. Another 3 mm long pick-up coil wound around the center of the wire was used to detect the magnetic flux of the amorphous wire. Figure 2 shows the B-H loop of the amorphous wire in its as-cast form and after annealing for 15 min. We could see the axial permeability was decreased by the annealing, and the magnetostriction value was also reduced by the annealing.

**Figure 3:**
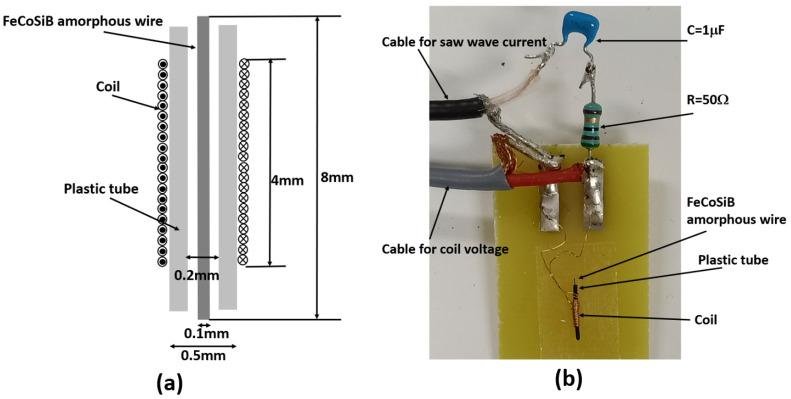
(**a**) Configuration of the coil with FeCoSiB amorphous wire as the coil core. (**b**) Photo of the coil.

For a normal inductive coil without or with a normal ferrite core, when an AC current flows into it, there is no DC voltage output for an applied DC magnetic field. A coil was made with a FeCoSiB amorphous wire as the core. Figure 3a shows the detailed configuration of the coil. A 40-turn coil was wound around a plastic tube using a ϕ 0.1 mm copper wire. The inner diameter of the plastic tube was 0.2 mm, and the outer diameter of the tube was 0.5 mm. The diameter of the FeCoSiB amorphous wire was 0.1 mm with a length of about 8 mm. The amorphous wire was put in the plastic tube. The length of the coil was about 4 mm. The coil with the FeCoSiB amorphous wire was fixed on a copper-removed PCB board using scotch tape. The dimensions of the coil and the amorphous wire were determined by considering the good coupling of the bias current and the easy fabrication of the coil. Figure 3b shows the photo of the coil.

**Figure 4:**
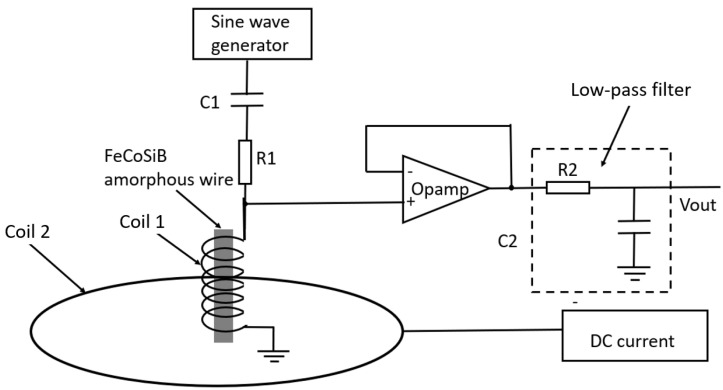
Block diagram of the setup to measure the DC voltage of the coil with FeCoSiB amorphous wire.

Figure 4 shows the block diagram of the measurements. Coil 1 shown in Figure 3 was connected to a signal generator which produced a high-frequency sine wave signal. The amplitude and the frequency could be adjusted. The capacitor C1 was 1 μF to isolate any possible DC currents from the signal generator, and the resistor R1 was 50 Ω. The operation amplifier of OP27 was used as a buffer with a gain of one. In this way, the measurement had less interruption in the voltage across the coil. The resistor R2 and the capacitor C2 composed a low-pass filter (LPF) to remove the high-frequency background signal. Additionally, the R2 = 300 Ω, and the C2 = 1 μF. The pass bandwidth of the LPF was from DC to 530 Hz. Coil 2 was connected to a DC current to produce a DC magnetic field. Coil 2 was 20 turns with a diameter of 20 cm. The size of coil 2 was much bigger than that of coil 1, so a near-uniform magnetic field was produced at the center of coil 2. Coil 1 and coil 2 were put into a three-layer permalloy magnetic shield box to reduce the influence of the environmental noise. The output voltage Vout was measured by an oscilloscope.

We measured the relations between the DC voltage of the coil and the DC magnetic field, and the frequency and the amplitude of the sine wave current were applied to the coil.

## 3. Results

### 3.1. DC Voltage Change with Applied DC Magnetic Field

**Figure 5:**
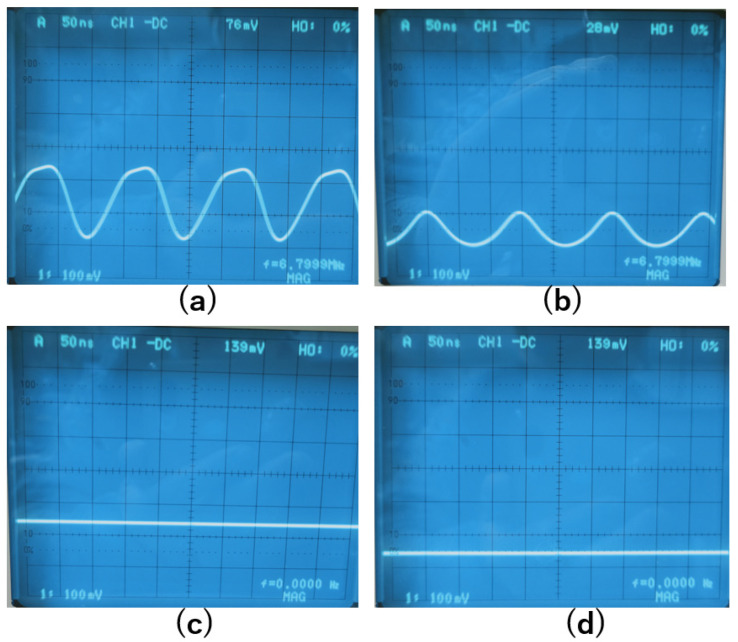
(**a**). Signal before the LPF without external magnetic field. (**b**). Signal before the LPF with 1 Gauss external magnetic field. (**c**). Signal after the LPF without external magnetic field. (**d**). Signal after the LPF with 1 Gauss external magnetic field.

The change of the DC voltage with the applied DC magnetic field when the AC current flowed into the coil was observed. In the beginning, the amplitude of the AC current was set to zero, and the DC output offset from the buffer amplifier was adjusted to zero. Then, the amplitude of the AC current was increased, and a DC voltage appeared even though there was no magnetic field applied, which was caused by the inherent magnetic field existing within the amorphous wire. When an external magnetic field was applied, the DC voltage output changed. The signals before and after the LPF were observed with an oscilloscope. Figure 5a,b show the signals before the LPF without and with an external magnetic field, respectively. The distortions of the signals were observed, and the change in the DC level was also observed when an external magnetic field was applied. Figure 5c,d show the signals after the LPF without and with an external magnetic field, respectively. After the LPF, the high-frequency signals were removed and only the DC voltages were left.

**Figure 6:**
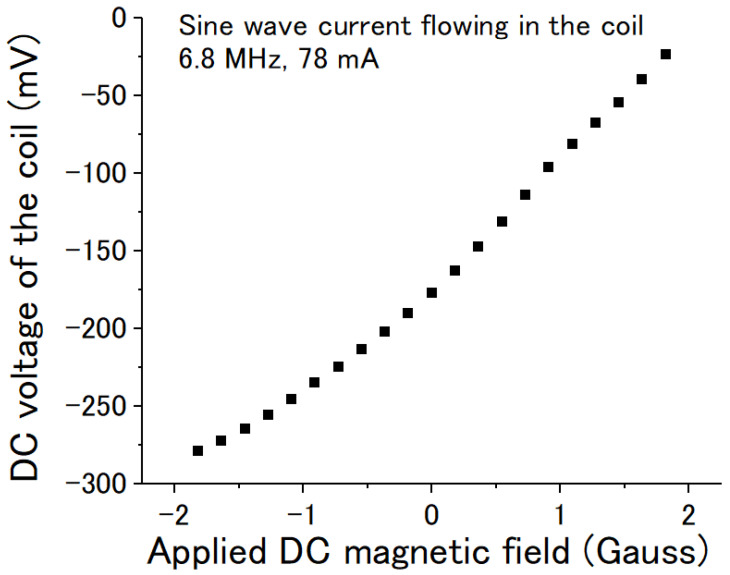
DC voltage of the coil vs the applied DC magnetic field. The sine wave current was 6.8 MHz with a peak-to-peak amplitude of about 78 mA.

Figure 6 shows the DC voltage output changing with the applied DC magnetic field when the frequency of the AC current was 6.8 MHz and when the peak-to-peak amplitude of the current was 78 mA. It was not exactly linear, but it increased with the DC magnetic field. The maximum transfer coefficient of the voltage/magnetic field ΔV/ΔB was estimated to be about 90 mV/Gauss. The voltage could be positive for different winding directions of the coil.

### 3.2. DC Voltage Change with the Frequency of the Sine Wave Current

**Figure 7:**
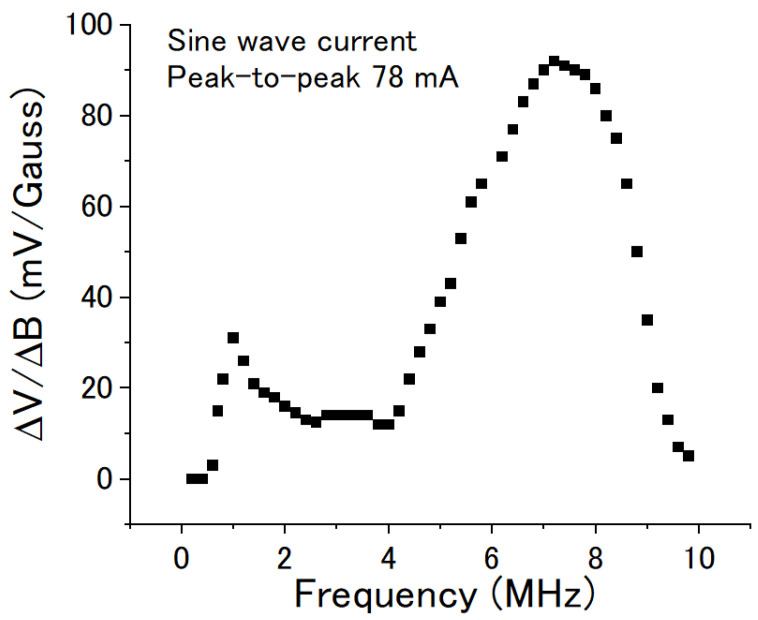
Voltage/field transfer coefficient ΔV/ΔB of the coil vs the frequency of the sine wave current. The peak-to-peak amplitude of the current was kept at 78 mA.

The DC voltage also changed with the frequency of the AC current at the same current amplitude. Figure 7 shows the voltage/field transfer coefficient ΔV/ΔB changing with the frequency of the sine wave current. The peak-to-peak amplitude of the AC current was kept at 78 mA. Two peaks were observed. One was at about 1 MHz, and another was at about 6.8 MHz. Additionally, a maximum ΔV/ΔB of about 90 mV/Gauss was obtained at about 6.8 MHz.

### 3.3. DC Voltage Change with the Amplitude of the Sine Wave Current

**Figure 8:**
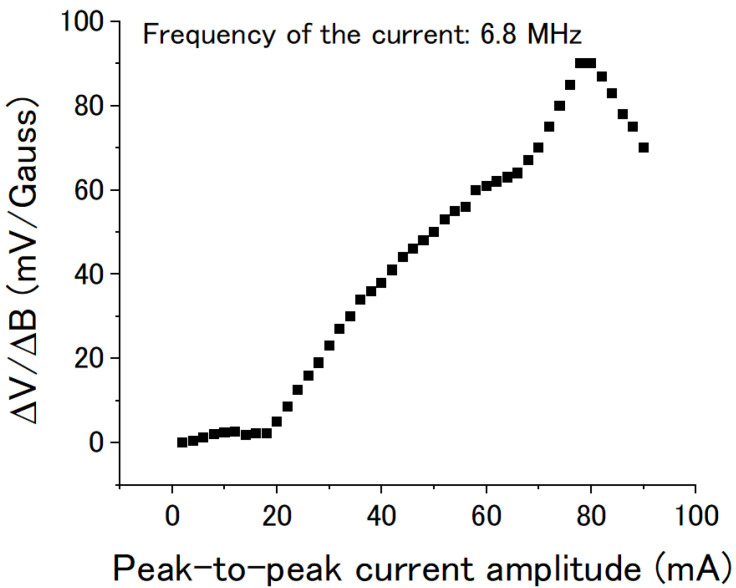
Voltage/field transfer coefficient ΔV/ΔB of the coil vs the peak-to-peak amplitude of the sine wave current. The frequency of the current was kept at 6.8 MHz.

The DC voltage also changed with the amplitude of the AC current at the same frequency. Figure 8 shows the voltage/field transfer coefficient ΔV/ΔB changing with the peak-to-peak amplitude of the sine wave current. The frequency of the AC current was kept at 6.8 MHz. The ΔV/ΔB increased rapidly when the current amplitude was over 20 mA, and a peak value of about 90 mV/Gauss was reached at about 78 mA. It then decreased with the amplitude of the AC current when the amplitude was over 80 mA.

The following hypothesis may explain the observed phenomenon: At the beginning, the magnetic domains in the amorphous wire point in one direction. The signal distortion is proportional to the amplitude of the AC current, so the DC voltage is also almost proportional to the amplitude of the AC current. However, if the amplitude of the AC current is greater than a critical value, the AC current will lead to a reversal of the direction of some magnetic domains, which will result in a decrease of the observed DC voltage.

### 3.4. Magnetic Sensor Based on the Nonlinear Effect of the Wire

**Figure 9:**
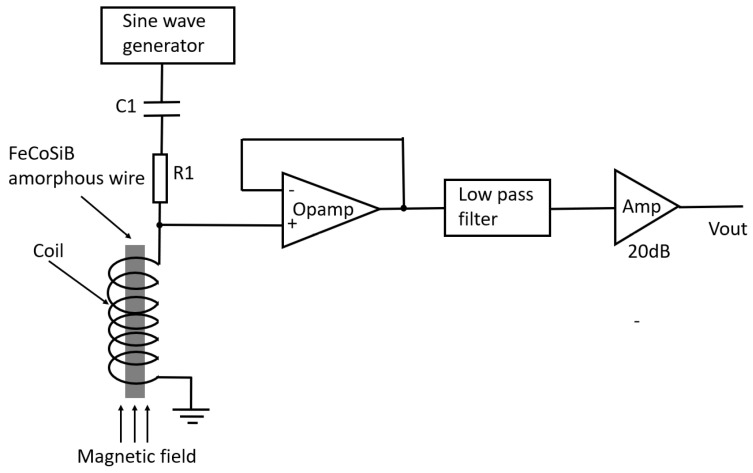
Schematic diagram of the magnetic sensor using the nonlinear effect of the coil with amorphous wire core.

Using the nonlinear effect of the amorphous wire, a novel type of magnetic sensor was developed. Figure 9 shows its schematic diagram. The driving circuit of the magnetic sensor was very simple. Only an AC bias current and an amplifier (Amp) were used. No demodulator was needed in the circuit.

The AC bias current was 6.8 MHz with a peak-to-peak amplitude of about 80 mA. The low-pass filter was used to remove the high-frequency signal caused by the AC bias current. The gain of the Amp was 20 dB. When an AC current with a 78 mA peak-to-peak amplitude flowed in the coil, the amplitude of the AC current was 39 mA. There were 40 turns of the coil, and it had a length of 4 mm. The amplitude of the magnetic field strength was about 390 A/m.

**Figure 10:**
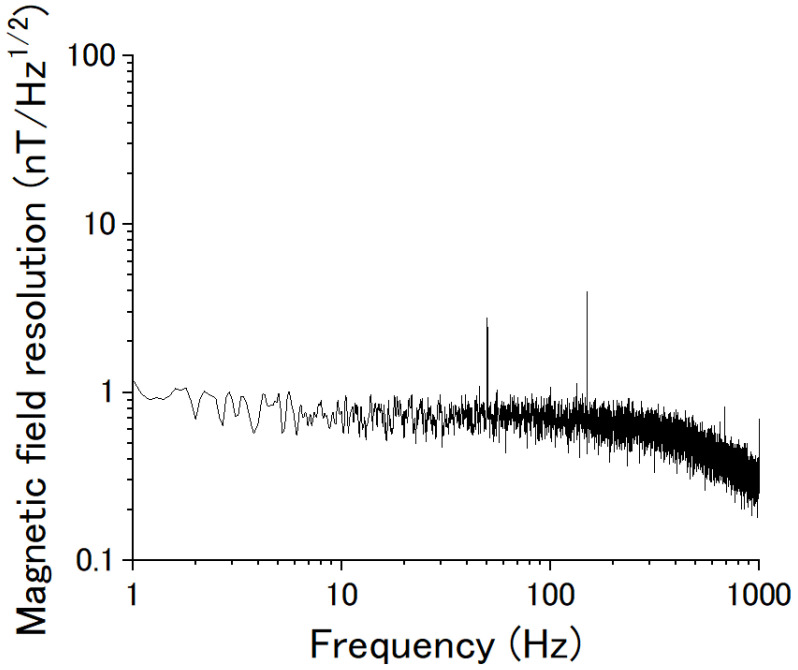
Magnetic field resolution of the novel type of magnetic sensor.

Figure 10 shows the magnetic field resolution of the magnetic sensor. As a preliminary result, a magnetic field resolution of about 1 nT/√Hz was obtained.

## 4. Discussion

The observed DC voltage had a relationship with the frequency and the amplitude of the AC current flowing through the coil. This effect was different from the GMI effect of the Co-rich amorphous wire. For the GMI effect measurement, the AC current was connected with the amorphous wire (shown in Figure 1a), and the circumferential magnetic field was produced. Additionally, for the GMI effect, the DC voltage change with the applied magnetic field was observed after demodulation. For the measurement of this effect, the AC current was connected to the coil (shown in Figure 4), and the parallel magnetic field was produced. Only an amplifier and a low-pass filter were used in the experimental setup. There was no demodulation circuit.

**Figure 11:**
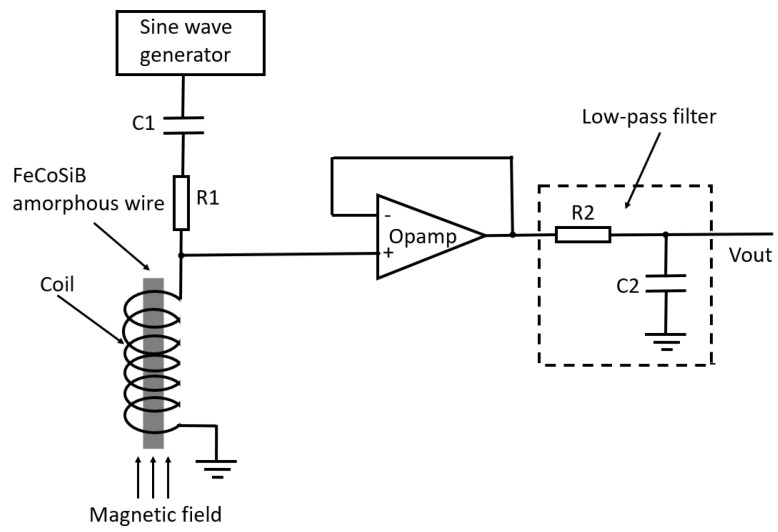
Experimental setup where a capacitor C3 was put between the coil and the Opamp.

In Figure 4, the coil was directly connected to the Opamp. To check the source of the abnormal DC voltage, a 1 μF capacitor C3 was inserted between the coil and the Opamp (shown in Figure 11) to isolate the DC voltage from the coil. However, the C3 had no influence on the experimental results shown in Figure 6, Figure 7 and Figure 8. The DC voltage change with the applied magnetic field was also observed before the amplifier using an oscilloscope, and the DC voltages were the same before and after the capacitor C3.

This “abnormal” phenomenon may have a relationship with the nonlinear effect of the FeCoSiB amorphous wire. When the coil with the amorphous wire core was biased by a large AC current, the nonlinear effect of the amorphous wire caused a signal distortion. The signal distortion was also influenced by the applied DC magnetic field. The distorted signal caused the DC output of the amplifier (or the amplifier in the oscilloscope). The skin effect also had an influence on the nonlinear effect of the amorphous wire, so the DC voltage also had relationship to the frequency of the AC current.

## Data Availability

Available upon requirement.
